# STAT2 hypomorphic mutant mice display impaired dendritic cell development and antiviral response

**DOI:** 10.1186/1423-0127-16-22

**Published:** 2009-02-19

**Authors:** Lan-Sun Chen, Pei-Chi Wei, Taming Liu, Chung-Hsuan Kao, Li-Mei Pai, Chien-Kuo Lee

**Affiliations:** 1 Graduate Institute of Immunology, National Taiwan University College of Medicine, 1 Jen-Ai Road, Section 1, Rm 513, Taipei 100, Taiwan, Republic of China; 2 Department of Biochemistry, Chang Gung University, Tao-Yuan, 333, Taiwan, Republic of China

## Abstract

Interferons (IFNs) are key regulators for both innate and adaptive immune responses. By screening ENU-mutagenized mice, we identified a pedigree- P117 which displayed impaired response to type I, but not type II, IFNs. Through inheritance test, genetic mapping and sequencing, we found a T to A point mutation in the 5' splice site of STAT2 intron 4–5, leading to cryptic splicing and frame shifting. As a result, the expression of STAT2 protein was greatly diminished in the mutant mice. Nonetheless, a trace amount of functional STAT2 protein was still detectable and was capable of inducing, though to a lesser extent, IFNα-downstream gene expressions, suggesting that P117 is a STAT2 hypomorphic mutant. The restoration of mouse or human STAT2 gene in P117 MEFs rescued the response to IFNα, suggesting that the mutation in STAT2 is most likely the cause of the phenotypes seen in the pedigree. Development of different subsets of lymphocytes appeared to be normal in the mutant mice except that the percentage and basal expression of CD86 in splenic pDC and cDC were reduced. In addition, *in vitro *Flt3L-dependent DC development and TLR ligand-mediated DC differentiation *were *also defective in mutant cells. These results suggest that STAT2 positively regulates DC development and differentiation. Interestingly, a severe impairment of antiviral state and increased susceptibility to EMCV infection were observed in the mutant MEFs and mice, respectively, suggesting that the remaining STAT2 is not sufficient to confer antiviral response. In sum, the new allele of STAT2 mutant reported here reveals a role of STAT2 for DC development and a threshold requirement for full functions of type I IFNs.

## Background

Interferon (IFN)s are classified into type I, II and III according to the receptors they bind to. Type I IFNs family consists of about 20 members of IFNα and only one member of IFNβ. The prominent function of type I IFNs is interfering viral infections through blocking viral transcription, degrading viral RNA, inhibiting translation and modifying proteins to control viral replication [1]. Other than antiviral responses, type I IFNs also exert different functions, including modulating innate and adaptive immunity, controlling cross presentation, promoting or impeding differentiation of certain cell types, and inhibiting cell proliferation [2,3]. Type II IFN consists of only one species of IFN- IFNγ that is mainly produced by T and NK cells when stimulated and is critical to their effector functions [4]. Other than regulating innate and adaptive immune response, type I and II IFNs also play important roles in cancer immunosurveillance and immunoediting, which place IFNs in a critical position in anticancer therapy [5].

Signal transduction of IFNs is mainly mediated by JAK-STAT pathway [6]. The binding of IFNα/β to IFNAR1 and IFNAR2 leads to activation of JAK1 and TYK2 followed by activation of STAT1, STAT2 and STAT3. STAT1, STAT2 and IRF9 form hetero-trimer, bind to interferon stimulated responsible element (ISRE) and transactivate downstream genes. STAT1 and STAT3 can also form homo- or hetero-dimers and bind to gamma activated site (GAS) to induce a different set of genes. Likewise, when IFNγ binds to IFNGR1 and IFNGR2, JAK1 and JAK2 are activated, which in turn activate STAT1. Activated STAT1 forms dimers and translocates into nucleus where the dimmer binds to GAS element in the promoter region of IFNg-inducible genes and trigger transactivation. The *in vivo *roles of STAT1, STAT2, and STAT3 have been well established by gene targeting in mice. Mice lacking STAT1 display severe impairment of antiviral responses [7,8]. Likewise, mice lacking STAT2 also exhibit similar phenotypes seen in STAT1KO mice [9], implying a pivotal role of STAT1 and/or STAT2 in response to viral infection. Other than impaired antiviral response, the functions of several immune cells including NK and B cells are also affected in mice lacking STAT1 [10,11]. Conventional knockout of STAT3 leads to early embryonic lethality, implying that STAT3 is developmentally indispensible [12]. However, using Cre-loxP system to induce STAT3 deletion in different tissues or organs unveils multiple functions of STAT3, ranging from cell survival, apoptosis, migration, development and differentiation [13-16].

In this study, we identified and characterized a pedigree of mutant mice (P117) which displayed impaired response to type I, but not type II, IFNs. A T to A point mutation in the 5' splice site of STAT2 intron 4–5 resulted in cryptic splicing and frame shift, leading to diminished production of STAT2 protein. Nonetheless, a small amount of STAT2 was detectable and remained functional, suggesting the STAT2 mutation results in hypomorphic and not null phenotype. *In vitro *and *in vivo *assays showed that DC development was defective in P117. Moreover, MEFs derived from P117 mice and the mice were extremely susceptible to viral infections, suggesting that the remaining STAT2 was not sufficient to confer antiviral response.

## Results

### Pedigree 117 displays impaired IFNα response

**Figure 1:**
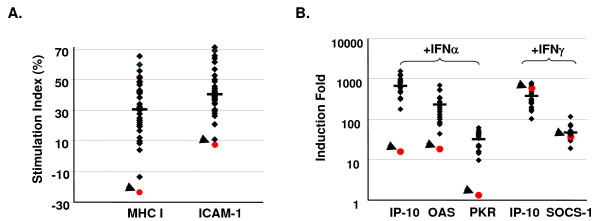
**P117 displayed impaired IFNα response**. (A) PBLs of ENU-mutagenized mice were stimulated without or with IFNα 1000 U/ml for 21 h followed by staining with antibodies to MHC class I and ICAM-1 and analyzed by flow cytometry. (B) PBLs of ENU-mutagenized mice were stimulated with IFNγ 500 ng/ml or IFNα 1000 U/ml for 3 h followed by preparation of mRNA for RT-QPCR using primers to IP-10 (IFNγ and IFNα), SOCS1 (IFNγ), OAS (IFNα) and PKR (IFNα). Stimulation index was calculated as described in the Materials and Methods. Relative mRNA was shown by normalizing the values of specific genes to that of β-actin. Arrow heads are P117.

Forward genetics or phenotype-driven approach using N-ethyl-N-nitrosourea (ENU) to induce mutations in mice provides a unique opportunity to assign functions to genes in an unbiased manner [17,18]. To screen ENU-mutagenized mice with defective IFN signaling, we designed a two-step screening method. The first step was to stimulate PBLs from ENU-mutagenized mice with IFNα or IFNγ, followed by measuring the surface expression of MHC class I and ICAM-1, molecules downstream of IFNs, by flow cytometry. Once deviant mice with enhanced or reduced IFN response were identified, a second confirmation step was performed. The PBLs of the deviant mice were stimulated with IFNs and the expressions of IP-10, OAS, PKR and SOCS1, four IFN-inducible genes were quantified by RT-QPCR. After screening some 2800 G3 mice from 134 pedigrees, we identified P117 as a poor IFNα responder to be of great interest. As shown in Fig. 1A, PBLs from P117 stimulated by IFNα expressed low levels of MHC class I and ICAM-1 as compared with wild-type controls. Likewise, the expressions of IFNα-inducible IP-10, OAS and PKR were also greatly reduced (Fig. 1B). However, IFNγ-induced IP-10 and SOCS-1 expressions were not affected in P117 mice (Fig. 1B). These results suggest that ENU-induced mutation in P117 is specific to IFNα signaling pathway.

### The mutation introduced to P117 is recessive and linked to Chromosome 10

**Figure 2:**
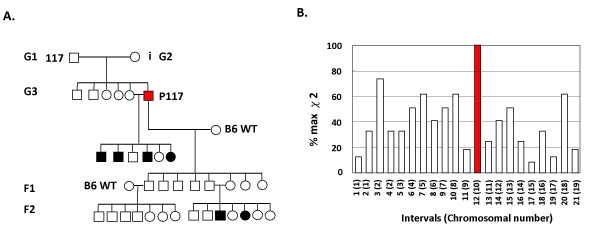
**Mutation in P117 was recessive and linked to chromosome 10**. (A) When P117 crossed with one of its siblings, a number of P117 offspring showed impaired IFNα response. When P117 crossed with wild-type mice, none of the F1 mice showed the phenotype. When the F1 mice intercrossed, the phenotype reappeared in the F2 mice. However, the phenotype was not observed in F2 mice of F1 crossed with wild-type mice. Empty squares and circles are unaffected males and females, respectively. Solid squares and circles are affected males and females, respectively. (B) Haplotype analysis was done as described in the Materials and Methods. The physical linkage of the mutation to chromosome in the mutant mice was based on the percentage of the maximum possible χ^2 ^(%max χ^2^). Solid bar: linked loci. Empty bars: unlinked loci.

To study if the reduced IFNα response in P117 was inheritable, P117 was first crossed with one of its siblings. Interestingly, a number of P117 offspring showed hyporesponsiveness to IFNα (Fig. 2A) which suggests the phenotype is inheritable. We next determined if the phenotype was recessive or dominant. P117 (in C57BL/6 background) was first crossed to wild type C57BL/6 mice. No aberrant response was observed in F1 mice. However, the phenotype reappeared in the F1-intercrossed F2 mice, suggesting that the aberrant IFNα response was recessive. To further confirm this finding, we crossed F1 mice with wild-type C57BL/6 mice and the phenotype disappeared in F2 mice. Together, the results of the inheritance test suggest that the altered IFNα response was recessive.

To identify that the mutation accounts for the aberrant response to IFNα, we first performed genetic mapping using interval haplotype analysis [19]. First, G3 affected male mouse was mated with C3H/HeJ females to generate F1 offspring that were then intercrossed to generate F2 mice. Of 292 F2 mice, 70 (~24%) were affected. DNA samples obtained from 15 individual affected F2 mice were subjected to interval haplotype analysis. The results are shown in Fig. 2B. Based on the percentage of maximum possible Chi square (%max χ^2^) value, linkage of the mutant gene was assigned to chromosome 10.

### STAT2 protein expression is diminished in P117

**Figure 3:**
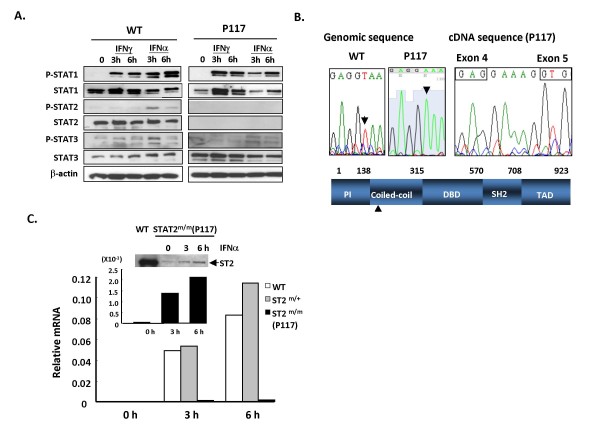
**A T to A point mutation in 5' splice site of the STAT2 intron 4–5 resulted in diminished expression of STAT2 protein in P117**. (A) Splenocytes of wild-type or P117 mice were stimulated with IFNγ or IFNα for 0, 3 and 6 h followed by Western blotting using antibodies to pSTAT1, STAT1, pSTAT2, STAT2, pSTAT3, STAT3 and β-actin. (B) Genomic sequencing results of wild-type (left panels) or P117 (middle panels). cDNA sequencing of p117 (right panels) indicated the addition of 5 nucleotides between exon 4 and exon 5. Schematic diagram of the mutation in the mouse STAT2 protein was shown (lower panel). PI: protein interaction, DBD: DNA binding domain, SH2: src homology 2, TAD: transactivation domain (C) Splenocytes of wild-type (empty bars), STAT2^+/m ^(gray bars) and STAT2^m/m ^(P117) (solid bars) mice were stimulated with IFNα (250 U/ml) for 0, 3 and 6 h, followed by RT-QPCR for OAS gene expression. Inset: response in STAT2^m/m ^(P117) cells in a smaller scale. Western blotting was done using antibody to STAT2. Arrow head indicated full length STAT2. Relative mRNA was shown by normalizing the values of OAS to that of β-actin.

Since P117 selectively displayed reduced response to IFNα but not IFNγ, we reasoned that the affected molecule might be one of the signal mediators specific to IFNα such as IFNAR1, IFNAR2, Tyk2, or STAT2. We first examined the expression of STATs and phospho-STATs in cells after stimulation with IFNs. Western blot analysis showed that while the protein levels of STAT1 and STAT3 were comparable in wild-type and P117 splenocytes, the STAT2 protein was not detectable in the mutant mice (Fig. 3A). Likewise, the levels of STAT1 and STAT3 tyrosine phosphorylation in P117 mice were also comparable with wild-type mice; yet IFNα-induced pSTAT2 (Tyr689) was completely undetectable in mutant cells. Immunohistochemical staining also showed that although STAT2 protein was expressed in different organs, including liver, kidney and the lung of wild-type mice, its expression in p117 was greatly reduced (data not shown).

To further examine whether the mutation was indeed harbored in the STAT2 gene, the STAT2 genomic DNA and cDNA were sequenced. We identified a T to A point mutation in the 5' splice site of intron 4–5 of the STAT2 gene in P117 (Fig. 3B left and middle panels). The point mutation was present in all the P117  that displayed altered IFNα response (data not shown). Mouse STAT2 gene encodes 923 amino acids in which 5 functional domains are defined, namely protein interaction (PI), coiled-coil, DNA binding (DBD), Src homology 2 (SH2), and transactivation domains (TAD) [20]. The mutation was located in the coiled-coil domain of STAT2 close to its N-terminal region (Fig. 3B lower panel). The point mutation resulted in cryptic splicing of STAT2 mRNA by using a new 5' splice site GU located three nucleotides downstream of the mutation (Fig. 3B, right panel). The alternative splicing caused frame-shift and created stop codons which left out critical domains of STAT2 protein including DBD, SH2 and TAD. To further confirm the phenotype seen in P117, we used RT-QPCR to examine induction of OAS gene by IFNα in splenocytes of wild-type, STAT2^+/m ^and STAT2^m/m ^(P117) mice. As shown in Fig. 3C, OAS mRNA expression was dramatically reduced in the mutant cells as opposed to STAT2^+/m^ or wild type control. Interestingly, however, we also noted that OAS gene expression in P117 cells was not completely diminished. Although the expression level following IFNα treatment for 6 h was approximately 1/40 of that in wild-type cells, it was still 20-fold higher than basal level of OAS in P117. These results suggest that a marginal IFNα response was still at work in P117 cells. In fact, at longer exposure, we observed a trace amount of full-length STAT2 protein in the mutant cells whose expression would increase further with time following IFNα treatment (Fig. 3C, inset). Therefore, we concluded that P117 mice were STAT2 hypomorphic instead of null mutant.

### Restoration of STAT2 rescues IFNα response in P117 MEFs

**Figure 4:**
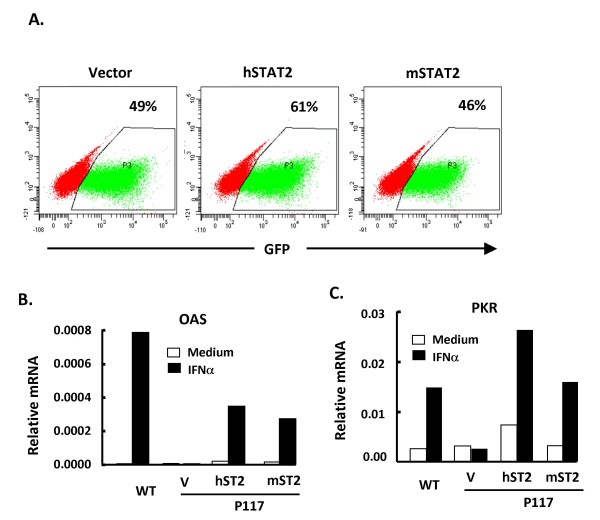
**STAT2 gene rescued IFNα response in P117 MEFs**. (A) P117 MEFs were infected with MigR1, MigR1-hSTAT2 or MigR1-mSTAT2 recombinant retrovirus. The infection efficiency was indicated by the percentage of GFP positive cells. P117 MEFs restored with vector alone (v), human STAT2 (hST2) or mouse STAT2 (mST2) gene were left untreated (medium, empty bars) or treated with 250 U/ml IFNα for 3 h (solid bars) followed by RT-QPCR using primers to OAS (B) or PKR (C). Relative mRNA was shown by normalizing the values of OAS or PKR to that of β-actin.

To confirm that IFNα hyporesponsiveness in P117 was due to mutation in STAT2 instead of elsewhere, we restored wild-type STAT2 back to P117 MEFs. It is reported that human and mouse STAT2 are divergent due to the presence of repetitive sequence in the C-terminus of the mouse STAT2 gene [20,21]. Therefore, bicistronic retroviruses carrying either wild-type human or mouse STAT2 gene and GFP were used to infect P117 MEFs. Judging from the percentage of GFP positive cells, the infection efficiency was between 46% and 61% (Fig. 4A). The infected cells were stimulated with IFNα followed by RT-QPCR to detect the induction of OAS and PKR mRNA. As shown in Fig. 4B and 4C, OAS and PKR were readily induced in control wild-type MEF after treatment. While transduction with vector alone did not alter IFNα hyporesponsiveness in the P117 MEF, restoration with hSTAT2 or mSTAT2 gene greatly rescued the inducibility of OAS and PKR by IFNα. These gain-of-function results suggest that the aberrant IFNα response in the mutant mice was due to defect in STAT2 expression.

### P117 displays impaired DC development in vivo and in vitro

**Figure 5:**
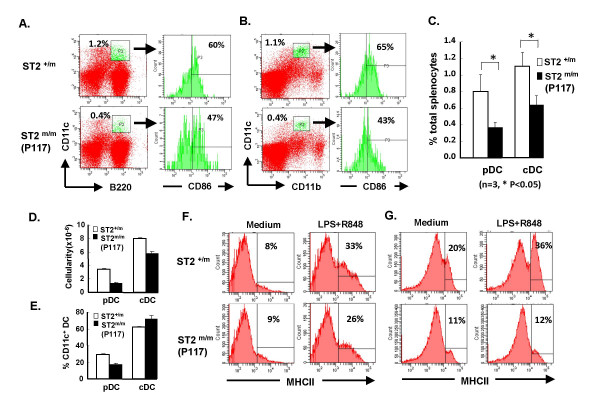
**Reduced percentage of DCs and impaired Flt3L-dependent DC development in P117**. Splenocytes of STAT2^+/m ^or STAT2^m/m ^(P117) mice were stained with antibodies to CD11c, CD86, and B220 or CD11b. The percentage of pDC (CD11c^+^B220^+^) (A) or cDC (CD11c^+^CD11b^+^) (B) in STAT2^+/m ^(upper panels) or STAT2^m/m ^(P117) (lower panels) and the percentage of CD86 ^high^ gated on these two populations were shown (right panels). (C) The average percentage of cDC and pDC of STAT2^+/m ^(empty bars) and STAT2^m/m ^(P117) (solid bars) mice was shown (n = 3). * P < 0.05  BM-derived  pDC and cDC from STAT2^+/m ^(empty bars) and STAT2^m/m ^(P117) (solid bars) mice were enumerated and analyzed by flow cytometry and the cellularity (D) and the percentage (E) were determined. pDC (F) and cDC (G) of STAT2^+/m ^(upper panels) and STAT2^m/m ^(P117) (lower panels) were then stimulated without (left panels) or with LPS plus R848 (right panels) for overnight and stained with anti-MHC class II, followed by flow cytometry.

To study whether STAT2 regulates hematopoiesis, cells from bone marrow, thymus, PBL and spleen of wild-type and P117 mice were stained and subjected to flow cytometry. The percentage and numbers of T cell subsets in the thymus, B cell subsets in the bone marrow and mature CD4, CD8, and B cells in the PBL and spleen in P117 mice were comparable to that in wild-type mice, suggesting that STAT2 does not regulate the development of T or B cells (data not shown). Since type I IFNs were reported to affect the maturation and functions of DC [22,23], we further tested if there was a defect in DC development in P117. As shown in Fig. 5A and 5B, left panels, the percentages of pDC (CD11c^+^B220^+^) and cDC (CD11c^+^CD11b^+^) were greatly reduced in the spleen of STAT2^m/m ^(P117) as opposed to STAT2^+/m ^mice and the difference was statistically significant (Fig. 5C). The cellularity of both types of DC was also reduced in the mutant (data not shown). Likewise, the expression of CD86 on STAT2^m/m ^(P117) pDC or cDC was also decreased (Fig. 5A and 5B, right panels).

It is reported that Flt3 signaling is important in DC development [24-26]. We first studied *in vitro *Flt3L-dependent DC development from BM. As shown in Fig. 5D and 5E, the numbers of pDC and cDC and the percentage of pDC from STAT2^m/m ^(P117) were lower than that from STAT2^+/m ^mice after Flt3L stimulation although the percentage of cDC from STAT2^m/m ^(P117) mice was not different from that of STAT2^+/m ^mice. Moreover, TLR ligand-induced MHC class II was lower in STAT2^m/m ^(P117) pDC than in STAT2^+/m ^pDC, although the basal level of MHC class II expression was comparable in STAT2^m/m ^(P117) and STAT2^+/m ^pDC (Fig. 5F). The effect of TLR ligands on MHC class II expression was even more pronounced in STAT2^m/m ^(P117) cDC. While the basal level of MHC class II expression was lower in STAT2^m/m ^(P117) cDC, its inducibility by TLR ligands was almost impaired (Fig. 5G). Experiments also show that the expression patterns of CD86 and ICAM-1 on STAT2^m/m ^(P117) pDC and cDC were similar to that of MHC class II before and after TLR-ligand stimulation (data not shown). However, the aberrant response was observed only when DC was derived from STAT2^m/m ^(P117) BM with Flt3L but not with GM-CSF stimulation (data not shown). These results suggest that STAT2 positively regulates Flt3L- but not GM-CSF -dependent DC development.

### P117 are sensitive to viral infection

**Figure 6:**
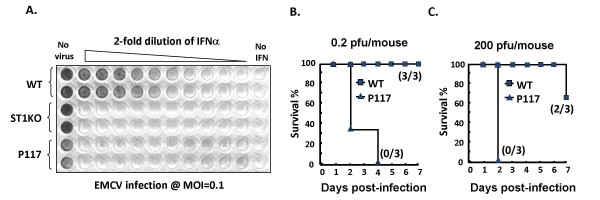
**Impaired antiviral response in P117 MEFs and mice**. (A) Wild-type, STAT1KO, and P117 MEFs were pretreated with 2-fold serial dilution of IFNα starting from 1000 U/ml for 24 h, followed by infection with EMCV at a M.O.I of 0.1. Live cells were visualized with crystal violet 18 h after the infection. Wild-type (squares) and P117 (triangles) mice were i.p. injected with 0.2 (B) or 200 (C) pfu/mouse of EMCV and survival rate was recorded every day for 7 days. Numbers in parenthesis represented live mice versus total mice at the end of the experiment.

It has been previously shown that mice completely lacking STAT2 were extremely susceptible to viral infections [9]. Since the P117 mice still expressed a trace amount of functional STAT2, we wondered if the remaining STAT2 could confer antiviral response. First of all, we did antiviral state assay *in vitro *by pretreating MEFs cells from wild-type, STAT1KO and P117 mice with 2-fold serial dilution of IFNα starting from 1000 U/ml followed by EMCV infection at M.O.I. of 0.1 and scored the lytic activity in MEFs. As shown in Fig. 6A, IFNα induced wild-type MEF antiviral state in a dose-dependent manner. However, IFNα even at 1000 U/ml failed to induce antiviral effect on either STAT1KO or P117 MEFs.

The impaired antiviral response *in vitro *was further confirmed *in vivo*. Wild-type and P117 mice were given  0.2 or 200 PFU of EMCV intraperitoneally. As shown in Fig. 6B, all P117 mice died within 2–4 days after infection with 0.2 pfu of EMCV, while all wild-type mice survived. The protective effect of STAT2 was obvious as only 1/3 of wild-type mice succumbed to 200 PFU of EMCV by day 7 (Fig. 6C). The LD_50 _of EMCV for P117 mice was, at least, 1000-fold lower than that of wild-type mice. Our work also showed that P117 mice were susceptible not only to EMCV but also to VSV infection (data not shown). These results indicate that P117 mice were extremely susceptible to viral infections and that the level of functional STAT2 expressed in P117 was not sufficient to protect mice from infection of different viruses.

## Discussion

We reported here the identification and characterization of the ENU-mutagenized mice p117 that displayed impaired response to type I, but not type II, IFNs. Inheritance test indicates that the deviant phenotype was recessive. Rough genetic mapping suggests that the mutation accounted for the phenotypes is linked to chromosome 10. Genomic and cDNA sequencing further confirmed a T to A point mutation located in the 5' splice site of intron 4–5 of STAT2 gene which leads to cryptic splicing and addition of 5 extra nucleotides between exon 4 and 5, causing frame shift and generation of stop codons. As a result, the expression of STAT2 protein was greatly diminished in the mutant mice. Three lines of evidence suggest that the mutation in STAT2 is highly likely the cause of the phenotypes seen in this pedigree. First of all, STAT2 only participates in type I, but not type II, IFN response. Secondly, STAT2 gene is located in the chromosome 10 which is in line with the genetic mapping. Thirdly, the restoration of human or mouse STAT2 in mutant cells rescues IFNα response.

Among the seven STAT family members in mice, STAT2 is relatively special compared to its human counterpart because of the insertion of a sequence containing 6 tandem copies of a 16 amino acid repeats at its C-terminus [20,21]. Because this sequence is not present in humans, it greatly reduces the homology between human and mouse to less than 40% in TAD domain. It was speculated that these repeats might mediate interactions with species-specific proteins that are important for the biology of STAT2. Indeed, Farrar et al. reported that type I IFNs induce Th1 differentiation in humans but not in mice by activating STAT4 and recruiting to the IFNα receptor complex via the C-terminus of STAT2. The insertion in mouse STAT2 alters the C-terminus and selectively disrupts its capacity to activate STAT4 [27]. However, this notion was negated by the finding that mice expressing a chimeric mouse STAT2 with human TAD domain were unable to activate STAT4 and trigger Th1 differentiation [28]. In fact, overexpression of mouse STAT2 in human U6 cell line that does not express STAT2 rescues IFNα response [21]. Here we also showed that the expression of human STAT2 in mouse P117 MEFs was able to correct the defect (Fig. 4). These data suggest that highly divergent primary sequences can serve similar functions and that the minimal regions of similarity between human and mouse STAT2 may define the critical regions for its function.

The roles of type I IFNs in DC development and maturation are still controversial. Hahm et al. reported that measles virus (MV) or lymphocytic choriomeningitis virus (LCMV) evades immune system by interfering DC development and expansion through generation of type I IFNs [29]. MV-infected mouse BM cells secrete soluble factors, including type I IFNs, *in vitro *to inhibit DC development from BM of wild-type or STAT1KO but not STAT2KO mice. Besides, LCMV cl13, which induces systemic type I IFN production during infection, interferes with DC expansion *in vivo *and reduces the frequency of DC progenitor cells within BM of wild-type, STAT1KO but not STAT2KO mice. These results suggest that type I IFNs negatively regulate DC development in a STAT2-dependent but STAT1-independent manner during viral infection. However, in IFNα receptor knockout mice (IFNARKO), the expression of costimulatory molecules or MHC class I and class II in GM-CSF-derived DC was reduced, so was the proliferating ability of T cells induced by the DCs [30]. Besides, mouse DCs undergo phenotypic maturation upon exposure to type I IFNs *in vitro *and *in vivo*. Recently, Trinchieri's group reported that the TLR ligand-mediated induction of CD40 and CD86 was greatly reduced in splenic pDC and cDC of IFNARKO mice compared to that of wild-type mice [31]. Since IFNα was induced in splenic DCs upon TLR ligand stimulation, these results also suggest that IFNα may participate in a positive feedback regulation of the maturation of DCs. Here, we showed a significant reduction of the percentages of splenic pDC and cDC in STAT2^m/m ^(P117) mice as opposed to STAT2^+/m ^or wild-type mice. Besides, *in vitro *assays also showed that Flt3L-dependent DC development and TLR ligand-mediated DC differentiation were also defective in cells from mutant mice (Fig. 5). In fact, gene profiling using DNA microarray has shown that human BDCA4^+ ^DC or mouse B220^+ ^plasmacytoid DC are among the highest expressers of STAT2 mRNA in different tissues and cells, indicating a physiological requirement of STAT2 for DCs [32]. Taken together, these results suggest a positively regulatory role of type I IFN and STAT2 in DC development.

Other than impaired IFNα response, P117 mice or cells were also sensitive to viral infections as indicated by reduced antiviral state *in vitro *and increased susceptibility to EMCV or VSV infection *in vivo *(Fig. 5 and data not shown). These phenotypes were very much reminiscent of STAT1KO or STAT2KO mice [7-9] in which type I IFN response is also impaired. There are similarities and differences between STAT2KO and P117 mice. In terms of gene expression, in general, the macrophages or splenocytes from both STAT2KO and P117 mice showed impaired response to type I IFNs while the response to type II IFN was intact. As for antiviral response, both types of mice are susceptible to VSV and EMCV infections, judging from increased viral burdens in STAT2KO mice and increased lethality in both mice. However, it still remains to be determined if the susceptibility to viral infection is comparable or not between STAT2KO and P117 mice. Nonetheless, there are also differences between these two systems. First of all, a trace amount of functional STAT2 is still present in P117 mice and it is capable of transducing signals to induce the expression of downstream OAS gene and STAT2 itself in response to IFNα (Fig. 3C). STAT2 protein is, however, completely devoid in STAT2KO mice and, therefore, there is a lack of ISGF3-mediated signaling in STAT2KO cells in response to IFNα. Secondly, basal level of STAT1 protein is reduced in STAT2KO MEF, which resulted in a block of type I IFN-dependent induction of MHC class I and impaired IFNγ-mediated antiviral response. We, however, did not observe any reduction of STAT1 protein in P117 MEF (data not shown) although the response to IFNγ has yet to be determined. The differential expression of basal level of STAT1 protein in these MEFs may lie in the presence of the residual STAT2 protein in P117 MEF, which may trigger a type I IFN-dependent autocrine loop to maintain the basal level of STAT1 expression in P117 cells [9,33-35]. Nonetheless, susceptibility of the mutant mice to viral infections also indicates that the residual STAT2 is not sufficient to mount an antiviral response and there is a threshold requirement of STAT2 for type I IFNs to be fully functional.

## Materials and methods

### Mice and cells

ENU-mutagenized mice were generated in the Mouse Mutagenesis Program Core Facility (MMPCF) at the Academia Sinica, Taiwan. The generation of ENU mice was done as described [36]. P117 mice were bred in C57BL/6J background. In this study, we use 6~8 week-old STAT2^m/m ^(P117), STAT2^+/m^, and wild-type littermate mice to do experiments. All mice are housed in the SPF environment at the NTUMC Animal Core Facility and the protocol was approved by the Institutional Animal Care and Use Committee at NTU. Generation of P117 embryonic fibroblasts (MEF) was done as described in the Current Protocols in Molecular Biology Unit 28.1. Primary MEFs were spontaneously immortalized by passaging the cells till they passed crisis. wild-type and STAT1KO MEF cell lines were provided by Dr. David Levy at New York University Medical Center Department of Pathology.

### Interval haplotype analysis

The interval haplotype analysis was done as described [19]. Briefly, genotype of a proximal and a distal marker for 21 chromosomal intervals distributed across the mouse genome (corresponding to two intervals for Chromosomes 1 and 2 and one for the remaining 17 autosomes) were used to do haplotyping. Male mice of P117 (C57BL/6 genetic background) were mated with C3H/HeJ females to generated F1 offspring that were then intercrossed to generate F2 mice. DNA samples obtained from 15 individual affected F2 mice were then subjected to interval haplotype analysis.

### Flow cytometry

Cells stimulated without or with IFNs were stained with fluorescence conjugated antibodies and subjected to flow cytometry analysis using FCASCanto (BD Biosciences). Antibodies used were the follows CD11b-FITC, CD11c-APC, B220-PE/FITC, CD86-PE/FITC, MHC I-FITC and ICAM-1-APC (all from e-bioscience). Stimulation index was calculated as follows (% MHC I^high ^or ICAM-1^high ^after treatment - % MHC I ^high ^or ICAM-1^high ^before treatment)/% MHC I ^high ^or ICAM-1^high ^before treatment.

### Real-time RT-QPCR

mRNA of PBL before and after IFN treatment was prepared using TurboCapture 96 mRNA kit (Qiagen) according to manufacturer's instructions. In some experiments, TRIzol reagent (Invitrogen) was used to prepare total RNA of splenocytes. One to three μg total RNAs prepared from cells treated with 250 U/ml IFNα (Merck) or 50 ng/ml IFNγ (Peprotech) for indicated times were subjected to reverse transcription. Quantitative real-time PCRs were carried out on the iCycler iQ real-time detection system (Bio-Rad). Each sample was performed in duplicates. The primer sequences used for PCR are the follows.

β-actin Forward 5'-AGGGAAATCGTGCGTGAC-3'

Reverse 5'-GCTCGTTGCCAATAGTGATG-3'

IP-10 Forward 5'-TGAGCAGAGATGTCTGAATCCG-3'

Reverse 5'-TGTCCATCCATCGCAGCA-3'

OAS Forward 5'-GCCATTGCACGCTCGCCTACTAC-3'

Reverse 5'-CTCCTGCCATCCGGGTTTTTCA-3'

PKR Forward 5'-TGCGCAGACAATGTATGGTAC-3'

Reverse 5'-ATGTGACAACGCTAGAGGATG-3'

SOCS1 Forward 5'-CCTGGTTGTAGCAGCTTGT-3'

Reverse 5'-CGACCCCTGGTTTGTGCAA-3'

### Western blotting

Total cell lysates of splenocytes or MEFs that were treated without or with IFN (IFNγ 50 ng/ml or IFNα 250 U/ml) for indicated times were subjected to electrophoresis. After transferring to nitrocellulose membrane, antibodies to tubulin (Sigma), phospho-STAT1 (tyrosine 701, Zymed), phospho-STAT2 (tyrosine 689, Upstate), and home-made rabbit antibodies to STATI and STAT2 were used for blotting.

### Retroviral transduction

Transduction of retroviruses into MEFs were done as described [37]. Briefly, a bicistronic retroviral vector containing mouse or human STAT2 cDNA and GFP was co-transfected with a plasmid expressing VSV G into 293T cells for 2 days before collecting the virus supernatant. MEFs were incubated with the virus supernatant in the presence of 8 μg/ml polybrene and spun at 1100 g for 90 min at 30°C. Flow cytometry were performed to measure GFP expression as an indicator of transduction efficiency in MEFs 2 days after the infection.

### In vitro culture of DCs

*In vitro *culture of DCs was done as described previously with slight modification [38]. Briefly, 1 × 10^7 ^BM cells were cultured in 10 ml of 10% FBS containing RPMI-1640 supplemented with 100 ng/ml mouse Flt3L (Peprotech), 50 μM β-ME (Sigma) and 50 μg/ml Gentamicin (Invitrogen) for 8 days. Different subsets of DCs were identified by staining the cells with anti-CD11c-APC, anti-CD11b-FITC and anti-B220-PE antibodies followed by flow cytometry. pDC (CD11c^+^CD11b^-^B220^+^) and cDC (CD11c^+^CD11b^+^B220^-^) were then sorted using FACSAria (BD Biosciences) and rested for 1 day before being stimulated with LPS 0.1 μg/ml plus R848 1 μg/ml for overnight and measured the expression of MHC class II using anti-I-A/I-E-PE antibody (Biolegend) to stain cells and measured by flow cytometry.

### In vitro and in vivo antiviral assays

For *in vitro *assay, wild-type, STAT1KO, or P117 MEFs were pretreated with 2-fold serial dilution of IFNα (1000 U/ml to 0.98 U/ml) for 24 h. EMCV at a M.O.I of 0.1 was added in serum free DMEM medium for 45 min 37°C. Viral supernatant was removed and the cells were refreshed with complete medium. Eighteen hours post-infection, medium was removed and cells were fixed with 10% formaldehyde solution for 20 min at RT. After fixation, cells were visualized with crystal violet. The excessive dye was then removed by immersing the plate in water. For *in vivo *assay, wild-type and P117 mice were infected with 0.2 or 200 pfu/mouse of EMCV by intraperitoneal injection. The survival rate was recorded every 24 h for 7 days.

### Statistics

*Student's T *test (two-tailed) was used for statistical analysis.

## Competing interests

The authors declare that they have no competing interests.

## Authors' contributions

LSC did the biochemical analysis, RT-QPCR and antiviral assays. PCW did the screening, inheritance test and identified the mutation. TL did the sequencing and flow cytometry. CHK did the sequencing and flow cytometry. LMP did immunohistochemical staining for tissue sections. CKL designed the experiments, helped to coordinate and drafted the manuscript.
